# Increased 30-day and 1-year mortality rates and lower coronary revascularisation rates following acute myocardial infarction in patients with autoimmune rheumatic disease

**DOI:** 10.1186/s13075-015-0552-2

**Published:** 2015-02-27

**Authors:** Sharon Van Doornum, Megan Bohensky, Mark A Tacey, Caroline A Brand, Vijaya Sundararajan, Ian P Wicks

**Affiliations:** Melbourne EpiCentre, The Royal Melbourne Hospital, Grattan Street, Parkville, Victoria 3050 Australia; The University of Melbourne, Grattan Street, Parkville, Victoria 3050 Australia

## Abstract

**Introduction:**

It is now well-recognised that patients with autoimmune rheumatic disease (AIRD) have a predisposition to cardiovascular disease that results in increased morbidity and mortality. Following myocardial infarction (MI), patients with rheumatoid arthritis have been shown to have an increased case fatality rate; however, this has not been demonstrated in other forms of AIRD. The aim of this study was to compare case fatality rates following a first MI in patients with AIRD versus the general population. The secondary aim was to compare revascularisation treatment following MI in patients with AIRD versus the general population.

**Methods:**

A retrospective cohort study using two population-based linked databases was undertaken. Cases of first MI from July 2001 to June 2007 were identified based on International Statistical Classification of Diseases and Related Health Problems, Tenth Revision, Australian Modification, codes. Thirty-day and one-year mortality rates were calculated (all-cause and cardiovascular causes of death). Logistic regression models were fitted to calculate the odds of mortality by AIRD status with adjustment for relevant characteristics.

**Results:**

There were 79,390 individuals with a first MI, of whom 1,409 (1.8%) had AIRD. After adjusting for relevant covariates, the odds ratio (OR) for 30-day cardiovascular mortality in patients with AIRD was 1.44 (95% confidence interval (CI): 1.25 to 1.66), and the OR for 12-month cardiovascular mortality was 1.71 (95% CI: 1.51 to 1.94). The 90-day adjusted odds of percutaneous transluminal coronary angioplasty and coronary artery bypass graft were significantly lower in the AIRD group compared with controls (OR: 0.81, 95% CI: 0.70 to 0.94, and OR: 0.52, 95% CI: 0.39 to 0.69, respectively).

**Conclusions:**

We identified a higher risk-adjusted mortality rate for the majority of patients with AIRD at 30 days and 12 months after first MI. We also identified lower post-MI revascularisation rates in the AIRD group, suggesting there may be current gaps in cardiovascular treatment for patients with AIRD.

**Electronic supplementary material:**

The online version of this article (doi:10.1186/s13075-015-0552-2) contains supplementary material, which is available to authorized users.

## Introduction

Diseases such as rheumatoid arthritis (RA), systemic lupus erythematosus (SLE), systemic sclerosis (SSc), spondyloarthritis (SpA) and systemic vasculitis have differing clinical presentations and immunopathogenic mechanisms, but they share the common feature of chronic systemic inflammation. These diseases, collectively referred to herein as *autoimmune rheumatic disease* (AIRD), also come with a predisposition to cardiovascular disease (CVD) [1]. Inflammation has been shown to be a critical aspect of the atherosclerotic process, influencing the development of endothelial dysfunction, plaque rupture and thrombosis [2]. Systemic inflammation in AIRD may drive the progression of underlying atherosclerotic disease, contributing to the increased CVD morbidity and mortality experienced by patients with AIRD [3-5].

A 2008 meta-analysis utilising data from 24 studies and 111,758 patients with RA (up to July 2005) reported a standardised mortality ratio from cardiovascular (CV) causes of 1.5 (95% CI: 1.39 to 1.61) [6]. This increased CV mortality can be partially explained by an increased incidence of myocardial infarction (MI) and other CVD [7-10]. However, we have demonstrated that patients with RA also have an increased case fatality rate following MI (adjusted odds ratio (OR) of 30-day mortality: 1.9 (95% CI: 1.3 to 2.7)) [11], a finding that has been replicated by others [9,12]. This increased case fatality rate may be partly explained by lower rates of post-MI treatment with acute revascularisation and/or cardioprotective drugs [13]. However, other possibilities include delays in the diagnosis of MI [14], the effect of concurrent medications (such as nonsteroidal anti-inflammatory drugs) [15,16], reduced physical activity and/or detrimental effects of systemic inflammation [17]. Patients with other forms of AIRD also have these potential risk factors and may also experience increased post-MI case fatality; however, this has not previously been investigated.

The primary aim of this study was to examine 30-day and 1-year mortality following a first acute MI in patients with AIRD versus the general population. The secondary aim was to compare revascularisation treatment (percutaneous transluminal coronary angioplasty (PTCA) and coronary artery bypass grafts (CABG)) following MI in patients with AIRD versus the general population.

## Methods

### Data sources

Two Australian population-based datasets, the Victorian Linked Dataset (VLD) and the Western Australian Data Linkage System (WADLS), which cover approximately 35% of the Australian population, were utilised [18]. Both datasets include hospital and emergency presentation data from all private and public hospitals across the state, along with linked national death data [19,20]. An evaluation of the WADLS linkage has shown that the extensive probabilistic matching procedures based on patient names and other partial identifiers are 99.89% accurate [20].

These datasets contain deidentified demographic and clinical information on each episode of patient care, with clinical information coded in the format of the International Statistical Classification of Diseases and Related Health Problems, Tenth Revision, Australian Modification (ICD-10-AM), beginning 1 July 1998 and the ICD Ninth Revision, Canadian Modification (ICD-9-CM), prior to 1 July 1998 [21]. The Victorian Department of Health maintains data quality with an annual independent audit cycle that samples nearly 1% of public hospital discharges. In the most recent audit, the sensitivity and positive predictive value of the hospital discharge coding for MI were 86% (95% CI: 80% to 93%) and 96% (95% CI: 92% to 100%), respectively, and for RA they were 91% (95% CI: 74% to 100%) and 77% (95% CI: 54% to 100%) [22]. The VLD provides place of residence information at the Australian census-derived Statistical Local Area (SLA) level, whereas the WADLS provides place of residence information at a postcode level.

### Definition of index myocardial infarction and autoimmune rheumatic disease

All cases of MI from 1 July 2001 to 30 June 2007 were identified based on the ICD-10-AM classifications (Additional file 1). An individual was considered to have experienced a first acute MI if she had a diagnosis of MI within this time period and no diagnosis of MI in the previous 5 years; that is, each dataset has records back to 1st July 1996 to provide a five-year ‘look-back’ period. A 5-year look-back period was chosen as this has been shown to exclude the vast majority of individuals who have experienced a prior MI, and it is likely that less than 5% of the remaining population have experienced an MI more than 5 years previously [23].

For the purposes of this study, AIRD included the following diagnoses: RA, SLE, psoriatic arthritis (PsA), ankylosing spondylitis (AS), enteropathic arthritis (EA), undifferentiated SpA, SSc, systemic necrotising vasculitis, Sjögren’s syndrome, polymyalgia rheumatica (PMR), mixed connective tissue disease (MCTD), dermatomyositis (DM) and polymyositis (PM). Each of these conditions was considered to be present when the relevant ICD-9-CM or ICD-10-AM diagnostic codes (see Additional file 1) were recorded during the index MI admission or during any admission in the 3 years prior to the index MI. We also aggregated PsA, AS, EA and undifferentiated SpA into a SpA group. We chose a 3-year look-back period for identifying AIRD after comparing different look-back periods in a sensitivity analysis. We found that the 3-year look-back provided optimal ascertainment of AIRD while still providing sufficient years of data to address our aims [24]. Patients who had more than one AIRD condition coded during the look-back period were considered to have AIRD and were therefore counted only once in the primary analysis.

### Outcomes

#### Mortality

Thirty-day and one-year mortality rates were calculated from the admission date of the index MI episode to the date indicated in the death registry data. Deaths occurring outside the hospital based on death reports to the death registry were also considered. For the VLD, cause of death was provided in a text format. To identify CV deaths, we used a text string-searching algorithm for the causes of death, allowing for permutations of the following words: myocardial infarction, angina pectoris, ischemic heart disease, congestive heart failure, cardiomyopathy, arrhythmias, pulmonary oedema, stroke, thromboembolism, and cardiac arrest. For the WADLS dataset, cause of death was provided in ICD-10-AM code format (Additional file 1).

#### Interventions in hospital

Procedure codes for PTCA and CABG were identified within the index MI episode or in any subsequent admission episode up to 90 days after the index MI to compare revascularisation rates for AIRD and non-AIRD patients (Additional file 1). As these procedures were not time- or date-stamped in the dataset, we were unable to identify the exact timing in relation to the MI event.

### Other covariates

Covariates in the adjusted models included age, sex, socioeconomic status (SES), proximity to goods and services and comorbidities of interest (based on the Charlson comorbidity algorithm [25,26]) (Additional file 1). We also included smoking, hypertension and hypercholesterolaemia in our analyses.

SES was indicated by the Index of Relative Socio-economic Disadvantage (IRSD), a measure included in the Socio-Economic Indexes of Areas (SEIFA) developed by the Australian Bureau of Statistics [27,28]. The IRSD is derived from census survey data related to disadvantage, such as low income, low educational attainment, unemployment and dwellings without motor vehicles. This index is thought to be most closely aligned with low SES and has been shown to be a predictor of poorer health outcomes and greater hospital utilisation [29]. A dichotomous variable reflecting residential areas in the lowest quartile (those with the greatest disadvantage) compared with those in the top three quartiles was generated from the IRSD.

Access to goods and services, including health care services, was determined by using the Accessibility/Remoteness Index of Australia (ARIA) measure [30]. This is an area-based measure based on each patient’s residential location. A dichotomized variable was generated to reflect those individuals whose place of residence was classified as ‘highly accessible’, indicating unrestricted access to goods and services, compared with those with restricted access.

### Statistical analysis

**Figure 1:**
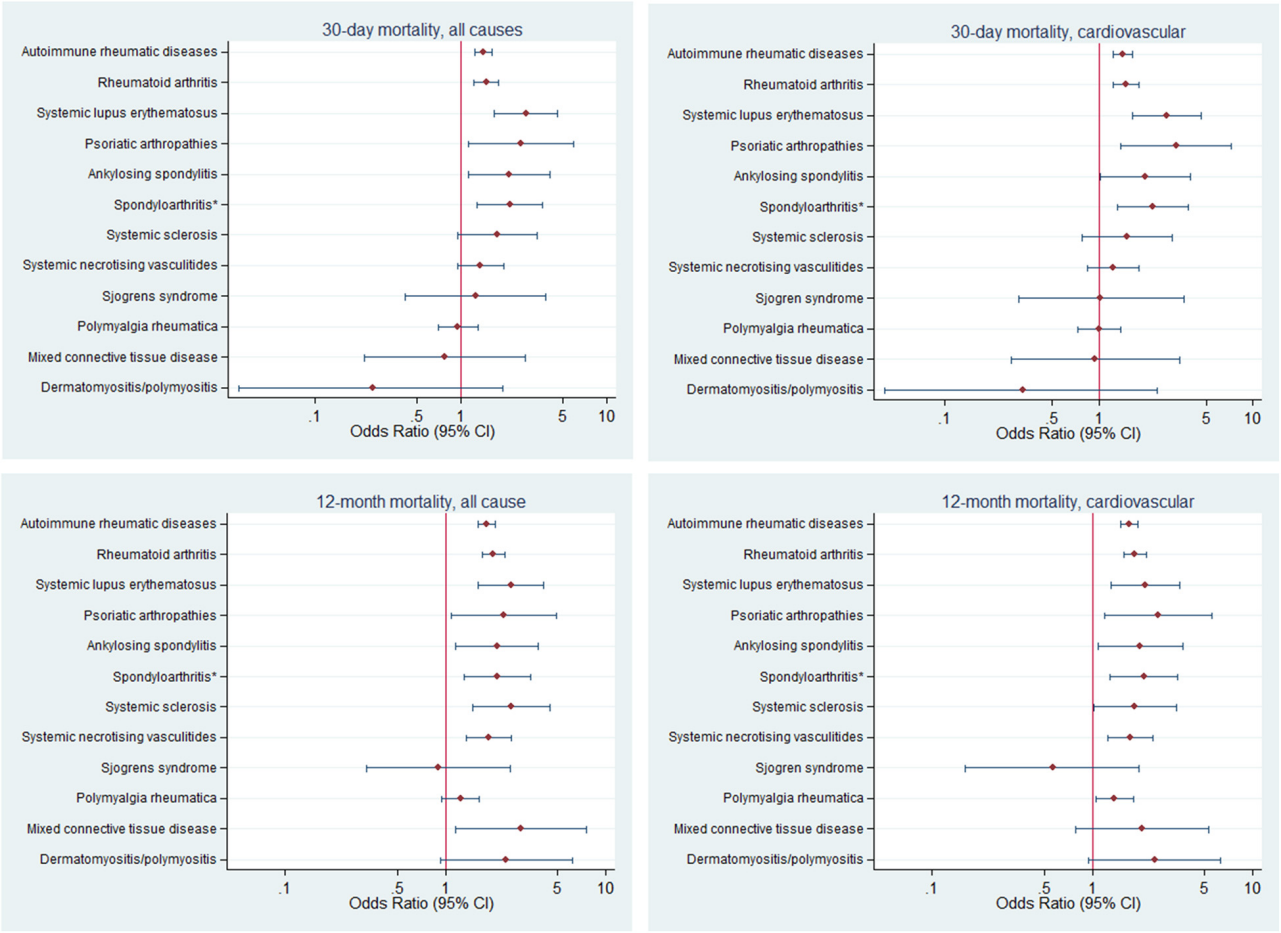
**Mortality risk for patients who experienced a first myocardial infarction between 1 July 2001 and 30 June 2007.** *Spondyloarthritis group includes ankylosing spondylitis, psoriatic arthritis, enteropathic arthritis and undifferentiated spondyloarthritis. Reference group is patients without autoimmune rheumatic disease (AIRD) or AIRD subgroup. For each AIRD subgroup, patients were excluded from the reference group if they had any other AIRD type. Adjusted for age, sex, Socio-Economic Indexes for Areas, Index of Relative Socio-economic Disadvantage, Accessibility/Remoteness Index of Australia high accessibility, individual Charlson comorbidities, smoking, hypertension and hypercholesterolaemia. ^95% CI, 95% confidence interval.

After initial descriptive statistics and exploratory bivariate analysis, logistic regression models were fitted with AIRD status as the key exposure variable and 30-day and 1-year mortality rates (all-cause and CV) as the outcome. Covariate adjustments included age, sex, IRSD, ARIA and relevant comorbidities (see Figure 1 for complete list). χ^2^ analyses were used to compare intervention rates in patients with versus without AIRD. Logistic regression analyses were used to examine the odds of receiving interventions by AIRD status after adjustment for age, sex, remoteness index, SES and comorbidities. Given the potential gain in efficiency of matching cases to controls, a sensitivity analysis was undertaken to explore this strategy. Non-AIRD controls were matched to AIRD cases on age and sex in a 5:1 ratio. All analyses were conducted using STATA statistical analysis software, version 12.1 (StataCorp, College Station, TX, USA). Ethical approval for this study was obtained from the Melbourne Health Human Research Ethics Committee. Individual patient consent was not required, as was determined by the ethics committee.

## Results

There were 79,390 individuals who experienced a first MI during the study period, of whom 1,409 (1.8%) had AIRD. The demographic and clinical characteristics of these patients are shown in Table 1 for the AIRD group overall and for the disease subgroups in Additional file 2. The patients with AIRD were older than those in the non-AIRD group (median (interquartile range) age: 77 years (68 to 83) versus 74 years (61 to 82)), more likely to be female (63.5% versus 39.1%) and less likely to be treated in public hospitals (63.7% versus 66.8%). Patients with AIRD were also more likely to have renal disease (20.2% versus 12.4%), pulmonary disease (11.4% versus 7.6%) and cerebrovascular accident (7.3% versus 5.4%). Patients with AIRD were less likely to have hypertension (41.1% versus 44.5%), a current history of smoking (11.9% versus 20.3%), hypercholesterolaemia (10.2% versus 16.4%) or cancer (4.7% versus 6.1%). The numbers of patients by AIRD subtype are reported in Table 2.

**Table 1:** **Patient demographic and clinical factors** ^**a**^

		**AIRD**	**Non-AIRD**
Patients, *n* (%)		1,409 (1.8)	77,981 (98.2)
Age, median (IQR)		77 (68 to 83)	74 (61 to 82)
Female, *n* (%)		894 (63.5)	30,489 (39.1)
Indigenous,^b^ *n* (%)		6 (2.0)	556 (3.7)
Married/de facto,^b^ *n* (%)		137 (46.3)	9,007 (59.5)
High accessibility to services,^c,d^ *n* (%)		1,232 (87.8)	67,550 (87.1)
Lowest quartile of SEIFA-IRSD, *n* (%)		190 (13.5)	11,102 (14.3)
Elective admission,^e^ *n* (%)		213 (19.1)	10,923 (17.4)
Public hospital admission,^e^ *n* (%)		709 (63.7)	41,965 (66.8)
Comorbidities at index admission, *n* (%)			
	Hypertension	579 (41.1)	34,687 (44.5)
	Arrhythmia	437 (31.0)	22,202 (28.5)
	Congestive heart failure	511 (36.3)	20,955 (26.9)
	Smoker	168 (11.9)	15,816 (20.3)
	Hypercholesterolaemia	143 (10.2)	12,750 (16.4)
	Diabetes	300 (21.3)	16,183 (20.7)
	Renal disease	284 (20.2)	9,649 (12.4)
	Pulmonary disease	161 (11.4)	5,927 (7.6)
	Cerebrovascular accident	103 (7.3)	4,227 (5.4)
	Cancer	67 (4.7)	4,781 (6.1)
	Obesity	47 (3.3)	3119 (4.0)
	Paraplegia	51 (3.6)	2,262 (2.9)
	Peripheral vascular disease	40 (2.8)	1,754 (2.3)
	Dementia	28 (2.0)	1,326 (1.7)
	Peptic ulcer disease	34 (2.4)	871 (1.1)
	Liver disease	16 (1.2)	443 (0.6)
	HIV	1 (<0.1)	30 (<0.1)

^a^AIRD, Autoimmune rheumatic disease; IQR, Interquartile range; IRSD, Index of Relative Socio-economic Disadvantage; SEIFA, Socio-Economic Indexes of Areas. ^b^Available for Western Australia data only (*n* = 15,426). ^c^Based on Accessibility/Remoteness Index of Australia (ARIA) code 1, Major Cities, Highly Accessible. ^d^Missing data for ARIA in Victoria data (*n* = 444). ^e^Available for Victoria data only (*n* = 63,964).

**Table 2:** **Number of autoimmune rheumatic disease conditions by subtype**

**Condition**	**Frequency,** ***n***	**%**
Rheumatoid arthritis	736	52.2%
Polymyalgia rheumatica	255	18.1%
Systemic necrotising vasculitis	177	12.6%
Systemic lupus erythematosus	100	7.1%
Systemic sclerosis	67	4.8%
Ankylosing spondylitis	59	4.2%
Psoriatic arthropathies	38	2.7%
Spondyloarthritis	95	6.7%
Dermatomyositis/polymyositis	25	1.8%
Mixed connective tissue disease	23	1.6%
Sjögren’s syndrome	22	1.6%
Total autoimmune rheumatic disease conditions, *N*	1,409	100%

### Mortality outcomes

The number of deaths in the AIRD and non-AIRD patients at 30 days and 12 months after the index MI, as well as the crude and adjusted ORs for mortality, are shown in Table 3. The 30-day all-cause mortality for AIRD and non-AIRD patients was 21.4% and 13.4%, respectively (*P* < 0.001). After adjusting for age, sex, SES, ARIA and relevant comorbidities, the OR for 30-day all-cause mortality in the AIRD group was 1.44 (95% CI: 1.26 to 1.65). The 30-day CV mortality for AIRD and non-AIRD patients was 18.9% and 11.7%, respectively (*P* < 0.001), with a corresponding adjusted OR of 1.44 (95% CI: 1.25 to 1.66). At 12 months, the patients with AIRD had an all-cause mortality rate of 38.6%, and that of non-AIRD patients was 22.8% (*P* < 0.001), with an adjusted OR of 1.82 (95% CI: 1.61 to 2.05). The 12-month CV mortality rates were 32.9% versus 19.4% in AIRD and non-AIRD patients, respectively (*P* < 0.001), with an adjusted OR of 1.71 (95% CI: 1.51 to 1.94). Figure 1 shows the adjusted ORs for the AIRD group overall, along with adjusted ORs for the disease subgroups that comprise the AIRD population. The adjusted ORs for the disease subgroups are also presented in Table 4. The adjusted ORs obtained in the sensitivity analysis using matched data were similar and therefore are not reported separately. They are available in Additional file 3.

**Table 3:** **Outcomes in patients with or without autoimmune rheumatic disease who experienced a first myocardial infarction between 1 July 2001 and 30 June 2007** ^**a**^

**Variable**	**Non-AIRD (n = 77,981)**	**AIRD (n = 1,409)**	**Crude OR (95% CI)**	**Adjusted OR** ^**b**^ **(95% CI)**
30-day mortality, all cause	10,452 (13.4)	301 (21.4)	1.76 (1.54 to 2.00)	1.44 (1.26 to 1.65)
30-day mortality, cardiovascular	9,105 (11.7)	266 (18.9)	1.76 (1.54 to 2.02)	1.44 (1.25 to 1.66)
12-month mortality, all cause	17,766 (22.8)	544 (38.6)	2.13 (1.91 to 2.38)	1.82 (1.61 to 2.05)
12-month mortality, cardiovascular	15,095 (19.4)	464 (32.9)	2.05 (1.83 to 2.29)	1.71 (1.51 to 1.94)

^a^AIRD, Autoimmune rheumatic disease; OR, Odds ratio. ^b^Adjusted for age, sex, Accessibility/Remoteness Index of Australia, Socio-Economic Indexes for Areas and comorbidities.

**Table 4:** **Adjusted odds ratios for the autoimmune rheumatic disease group overall and disease subgroups** ^**a**^

**Condition**	**Count**	**30-day mortality (adjusted odds ratio** ^**b**^ **)**		**12-month mortality (adjusted odds ratio** ^**b**^ **)**	
		**All causes**	**Cardiovascular**	**All causes**	**Cardiovascular**
Autoimmune rheumatic diseases	1,409	**1.44 (1.26 to 1.65)**	**1.44 (1.25 to 1.66)**	**1.82 (1.61 to 2.05)**	**1.71 (1.51 to 1.94)**
Rheumatoid arthritis	666	**1.50 (1.24 to 1.81)**	**1.51 (1.24 to 1.84)**	**2.00 (1.69 to 2.35)**	**1.85 (1.57 to 2.19)**
Systemic lupus erythematosus	93	**2.81 (1.71 to 4.62)**	**2.77 (1.65 to 4.64)**	**2.57 (1.60 to 4.13)**	**2.14 (1.30 to 3.51)**
Psoriatic arthropathies	25	**2.61 (1.14 to 5.97)**	**3.19 (1.40 to 7.27)**	**2.33 (1.09 to 4.97)**	**2.59 (1.19 to 5.63)**
Ankylosing spondylitis	38	**2.14 (1.13 to 4.08)**	**2.01 (1.02 to 3.95)**	**2.11 (1.16 to 3.82)**	**1.98 (1.08 to 3.66)**
Spondyloarthritis^c^	63	**2.19 (1.30 to 3.67)**	**2.26 (1.32 to 3.86)**	**2.11 (1.31 to 3.39)**	**2.10 (1.29 to 3.42)**
Systemic sclerosis	61	1.78 (0.95 to 3.33)	1.53 (0.78 to 3.03)	**2.58 (1.48 to 4.48)**	**1.85 (1.03 to 3.34)**
Systemic necrotising vasculitides	174	1.37 (0.95 to 1.98)	1.24 (0.84 to 1.84)	**1.87 (1.35 to 2.60)**	**1.73 (1.25 to 2.41)**
Sjögren’s syndrome	17	1.27 (0.42 to 3.86)	1.03 (0.30 to 3.57)	0.90 (0.32 to 2.53)	0.57 (0.16 to 1.97)
Polymyalgia rheumatica	239	0.96 (0.70 to 1.32)	1.01 (0.73 to 1.40)	1.25 (0.95 to 1.63)	**1.38 (1.05 to 1.81)**
Mixed connective tissue disease	19	0.78 (0.22 to 2.76)	0.95 (0.27 to 3.36)	**2.96 (1.16 to 7.56)**	2.06 (0.79 to 5.37)
Dermatomyositis/polymyositis	25	0.25 (0.03 to 1.95)	0.32 (0.04 to 2.42)	2.40 (0.93 to 6.19)	2.45 (0.95 to 6.31)

^a^Statistically significant results in bold. ^b^Adjusted for age, sex, Accessibility/Remoteness Index of Australia, Socio-Economic Indexes for Areas and comorbidities. ^c^Spondyloarthritis group includes ankylosing spondylitis, psoriatic arthritis, enteropathic arthritis and undifferentiated spondyloarthritis.

### Interventions

Rates of PTCA within the index admission were significantly lower in the AIRD patient group than in the non-AIRD patient group (AIRD 13.5% versus non-AIRD 25.9%, *P* < 0.001, crude OR: 0.53 (95% CI: 0.46 to 0.62)) (Table 5). This difference persisted after adjustment for age, sex, ARIA, SEIFA and comorbidities (adjusted OR: 0.80 (95% CI: 0.68 to 0.94)). The intervention rates of CABG within the index admission were also significantly less in the AIRD patient group (2.5% versus 5.9%, *P* < 0.001, crude OR: 0.41 (95% CI: 0.29 to 0.57)), which persisted after adjustment (adjusted OR: 0.50 (95% CI: 0.35 to 0.70)). In those who did not undergo intervention during their index admission, intervention rates were higher in the non-AIRD patient group than in the AIRD patient group at 30 days and between 30 and 90 days, although this was not significant for CABG at 30 days. Following adjustment, there were no differences in intervention rate at either 30 or 90 days.

**Table 5:** **Interventions in patients with or without autoimmune rheumatic disease who experienced a first myocardial infarction between 1 July 2001 and 30 June 2007** ^**a**^

**Variable**		**Non-AIRD (** ***n*** **= 77,981)**	**AIRD (** ***n*** **= 1,409)**	***P*** **-value**	**Crude odds ratio (95% CI)**	***P*** **-value**	**Adjusted odds ratio** ^**b**^ **(95% CI)**	***P*** **-value**
PTCA								
	Within index MI admission	17,622 (22.6)	190 (13.5)	<0.001	0.53 (0.46 to 0.62)	<0.001	0.80 (0.68 to 0.94)	0.007
	Postdischarge and within 30 days of index MI episode	1,355 (2.2)	17 (1.4)	0.046	0.62 (0.36 to 0.99)	0.046	0.90 (0.56 to 1.44)	0.652
	Between 30 and 90 days of index MI episode	1,192 (2.0)	14 (1.2)	0.036	0.57 (0.31 to 0.97)	0.036	0.95 (0.55 to 1.66)	0.860
CABG								
	Within index MI admission	4,592 (5.9)	35 (2.5)	<0.001	0.41 (0.29 to 0.57)	<0.001	0.50 (0.35 to 0.70)	<0.001
	Postdischarge and within 30 days of index MI episode	809 (1.1)	9 (0.7)	0.114	0.59 (0.27 to 1.13)	0.114	0.80 (0.43 to 1.51)	0.498
	Between 30 and 90 days of index MI episode	1,350 (1.9)	9 (0.7)	0.001	0.35 (0.16 to 0.67)	0.001	0.59 (0.30 to 1.15)	0.120

^a^AIRD, Autoimmune rheumatic disease; CABG, Coronary artery bypass graft; CI, Confidence interval; MI, Myocardial infarction; PTCA, Percutaneous transluminal coronary angioplasty. ^b^Adjusted for age, sex, Accessibility/Remoteness Index of Australia, Socio-Economic Indexes for Areas and Charlson comorbidities.

## Discussion

To our knowledge, this is the largest study of case fatality rates following MI in patients with AIRD. Patients with AIRD experiencing a first MI had adjusted odds of death that were 1.5 times greater than that for patients without AIRD at 30 days (OR: 1.44, 95% CI: 1.26 to 1.65) and nearly two times that of patients without AIRD at 12 months (OR: 1.82, 95% CI: 1.61 to 2.05). Post-MI mortality was also significantly increased in most subgroups, including RA, SLE, PsA and AS (at 30 days and 12 months) and SSc, vasculitis, PMR and MCTD at 12 months. Sjögren’s syndrome was the only AIRD that did not demonstrate an association with mortality after MI. This may be due to the small number of patients (*n* = 22), as there is some evidence to suggest cardiac involvement in these patients [31]. Although dermatomyositis/polymyositis showed a positive association with mortality after MI at 12 months, the association was not significant. This also may be related to the small number of patients in this group (*n* = 25).

There have been relatively few studies of post-MI case fatality rates in patients with AIRD. Three studies in RA populations have all demonstrated increased case fatality rates following MI [9,11,12], whereas the results of two studies of in-hospital mortality after MI in lupus patients were inconsistent. In a comparison of outcomes after acute MI in 519 SLE patients and 214,771 controls in a California hospitalisation database, in-hospital mortality was not found to differ between the groups [32]. In contrast, a similar analysis using the 1993–2002 US Nationwide Inpatient Sample reported a hazard ratio (HR) of 1.65 (95% CI: 1.33 to 2.04) for in-hospital mortality in SLE [33].

We also identified disparities in intervention rates between AIRD and non-AIRD patients within the index admission. We recently reported similar findings in a study of 90 patients with RA drawn from three Australian hospitals. In that study, we identified lower rates of acute reperfusion in the RA group [13]. Similarly, in a retrospective cohort study in the United States, researchers reported lower rates of CABG (HR: 0.35, 95% CI: 0.16 to 0.78) in a comparison of 1,206 age- and sex-matched patients with RA and controls [14]. In contrast to these findings, McCoy *et al.* reviewed medical records from 1979 to 2009 in the United States, including 77 patients with RA, and identified no differences in intervention rates, including thrombolytic therapy, PTCA and CABG [34]. Francis *et al.* conducted a cross-sectional analysis of 1,112,676 patients with MI in the United States and found that patients with RA (*n* = 13,029) were 38% (95% CI: 10% to 71%) more likely to receive thrombolysis and 27% (95% CI: 17% to 39%) more likely to receive PTCA [35]. As patients with RA comprised the majority of the cohort in our study (52%) and shared similar demographic characteristics with the aforementioned studies, the differences in treatment patterns may be related to practice variation in different geographical areas. We did not identify any studies in which treatment patterns in other (non-RA) AIRD populations were examined.

The patients with AIRD in this study tended to be older, were more likely to be female and/or unmarried and had significantly higher rates of certain comorbidities, including renal disease, pulmonary disease, peptic ulcer disease, cerebrovascular accident and liver disease than non-AIRD patients. As some of these are known risk factors for mortality and morbidity following coronary intervention [36], clinicians may have been more reluctant to recommend invasive procedures for this patient group. As we did not have detailed clinical data on cardiac risk factors or clinician decision making, we are not able to determine whether these factors influenced intervention rates. Interestingly, patients with AIRD had significantly lower rates of certain CV risk factors compared with non-AIRD patients, including smoking, hypertension and dyslipidaemia. This finding is consistent with previous studies that have identified a clinical presentation of CVD in patients with RA that is different from traditional CVD presentation patterns, which may delay disease recognition [11,14] and/or treatment [13].

Current guidelines recommend CV screening for patients with RA and consideration of monitoring patients with AS and PsA [37]. Given the relationship between inflammatory processes and CVD, examining biomarkers of inflammation and the presence of AIRD conditions may be warranted as part of existing CVD risk prediction models. Identifying inflammatory markers has been shown to be effective for detecting non-AIRD patients at high risk of CV events. The findings of the JUPITER trial, in which researchers examined the use of rosuvastatin as primary prevention therapy to reduce CV events in people with elevated high-sensitivity C-reactive protein, support the use of inflammatory biomarkers in assessing CV risk [38]. A further study is underway to examine whether inhibiting inflammation with methotrexate is effective for reducing adverse CV outcomes [39]. Further research should consider whether anti-inflammatory treatments in patients with AIRD will contribute to reductions in CV risk and mortality.

A major strength of our study is that we utilised two population-based datasets with coverage of 35% of Australia’s population, including hospital admissions, emergency department presentations and deaths outside the hospital. However, we must also acknowledge several limitations. We utilized administrative datasets that are not designed primarily for research purposes. Clinical information in these types of data has been shown to have inaccuracies [40], and our estimates of CV risk factors may be inaccurate. This risk of error is unlikely to be differentially distributed between the groups, and therefore the risk of introduction of bias is low. There may be a bias towards coding more severe cases of AIRD, which would compromise the generalisability of our findings. Our finding of increased CV mortality across many of the AIRD subgroups is consistent with the hypothesis of systemic inflammation contributing to adverse CV outcomes; however, our data cannot address this specifically. The various disease subgroups have different aetiopathogenic mechanisms and treatment approaches that may increase CV risk via independent pathways. For instance, thrombosis may play a more important role in patients with SLE, whereas pulmonary hypertension and diastolic heart failure may be more relevant in patients with SSc. We found thrombolysis to be undercoded, as drug therapies are not routinely captured in these data, so thrombolysis rates could not be included in our analyses. We also used data from two different Australian states, which had different methods of coding some variables. A number of variables could not be analysed because of these differences, and our analysis of causes of death required different methodologies. However, comparisons between the two states revealed no significant differences in the distributions of causes of death. We chose a 5-year look-back period to identify a history of previous MI. Although this may have misclassified some subsequent MIs as the first MI, an individual who experienced MI during the study period and had a history of previous MI more than 5 years prior would have been likely to receive treatment comparable to that of individuals with a true first MI [41].

## Conclusions

We have demonstrated increased 30-day and 12-month case fatality rates following an incident MI in patients with a range of AIRD conditions compared with the general population after adjustment for age, sex, SES, regional residence and significant comorbidities. We also identified lower rates of PTCA and CABG in the AIRD group within the index admission. Further studies are required to determine whether the management of patients with AIRD post-MI differs from that of non-AIRD patients. In future research, investigators should examine the use of inflammatory markers for identifying patients with AIRD who are at high risk and the influence of anti-inflammatory therapy on CV risk in patients with AIRD.

## Additional files

Additional file 1:
**ICD-9-CM and ICD-10-AM classification codes for autoimmune rheumatic disease, myocardial infarction, co-morbidities and procedures.**
Additional file 2:
**Demographic and clinical characteristics for autoimmune rheumatic disease overall and for each disease subgroup.**
Additional file 3:
**Results of matched case–control (1:5 ratio) analysis.** Table S1 Patient demographic and clinical factors, based on case–control analysis. Table S2 Outcomes in patients with versus without autoimmune rheumatic disease (AIRD) who experienced a first MI between 1 July 2001 and 30 June 2007, based on case–control analysis.

## Abbreviations

AIRD: Autoimmune rheumatic disease
ARIA: Accessibility/Remoteness Index of Australia
AS: Ankylosing spondylitis
CABG: Coronary artery bypass graft
CI: Confidence interval
CV: Cardiovascular
CVD: Cardiovascular disease
DM: Dermatomyositis
EA: Enteropathic arthritis
HR: Hazard ratio
ICD-10-AM: International Statistical Classification of Diseases and Related Health Problems, Tenth Revision, Australian Modification
ICD-9-CM: International Statistical Classification of Diseases and Related Health Problems, Ninth Revision, Canadian Modification
IQR: Interquartile range
IRSD: Index of Relative Socio-economic Disadvantage
MCTD: Mixed connective tissue disease
MI: Myocardial infarction
OR: Odds ratio
PM: Polymyositis
PMR: Polymyalgia rheumatica
PsA: Psoriatic arthritis
PTCA: Percutaneous transluminal coronary angioplasty
RA: Rheumatoid arthritis
SEIFA: Socio-Economic Indexes for Areas
SES: Socioeconomic status
SLA: Statistical Local Area
SLE: Systemic lupus erythematosus
SpA: Spondyloarthritis
SSc: Systemic sclerosis
VLD: Victorian Linked Dataset
WADLS: Western Australian Data Linkage System
