# Supplementary material for: Increased 30-day and 1-year mortality rates and lower coronary revascularisation rates following acute myocardial infarction in patients with autoimmune rheumatic disease
Source: Arthritis Res Ther. 2015 Feb 27;17(1):38. doi: 10.1186/s13075-015-0552-2 (PMC4372281; doi:10.1186/s13075-015-0552-2)
Supplement: Additional file 2: — Demographic and clinical characteristics for autoimmune rheumatic disease overall and for each disease subgroup. [file 13075_2015_552_MOESM2_ESM.doc]

**Appendix 2 – Demographic and clinical characteristics for AIRD overall and each disease subgroup** (web only)^

| **Variable** | **Non-AIRD** | **AIRD** | **RA** | **PMR** | **SNV** | **SLE** | **SSc** | **AS** | **PsA** | **DMPM** | **MCTD** | **SjS** |  | |
| --- | --- | --- | --- | --- | --- | --- | --- | --- | --- | --- | --- | --- | --- | --- |
|  | |
|  | |
| Patients, n (%) | 77,981 (98.2) | 1,409 (1.8) | 736 | 255 | 177 | 100 | 67 | 59 | 38 | 25 | 23 | 22 |  | |
| Age, med (IQR) | 74 (61 - 82) | 77 (68 - 83) | 76 (68 - 82) | 82 (78 - 87) | 78 (72 - 84) | 70 (54 - 77) | 71 (60 - 79) | 72 (63 - 80) | 68 (57-76) | 70 (62 - 80) | 70 (67 - 83) | 76 (72 - 78) |  | |
| Female, n (%) | 30,489 (39.1) | 894 (63.5) | 474 (64.4) | 172 (67.5) | 101 (57.1) | 74 (74.0) | 51 (76.1) | 25 (42.4) | 22 (57.9) | 12 (48.0) | 14 (60.9) | 16 (72.7) |  | |
| Hypertension | 34,687 (44.5) | 579 (41.1) | 294 (40.0) | 114 (44.7) | 81 (45.8) | 41 (41.0) | 25 (37.3) | 30 (50.9) | 13 (34.2) | 10 (40.0) | 6 (26.1) | 8 (36.4) |  | |
| Heart Failure | 20,955 (26.9) | 511 (36.3) | 273 (37.1) | 106 (41.6) | 63 (35.6) | 26 (26.0) | 25 (37.3) | 21 (35.6) | 13 (34.2) | 6 (24.0) | 11 (47.8) | 14 (18.2) |  | |
| Smoker | 15,816 (20.3) | 168 (11.9) | 102 (13.9) | 13 (5.1) | 14 (7.9) | 19 (19.0) | 6 (9.0) | 7 (11.9) | 5 (13.2) | 3 (12.0) | 4 (17.4) | 0 |  | |
| Hypercholesterolaemia | 12,750 (16.4) | 143 (10.2) | 65 (8.8) | 30 (11.8) | 22 (12.4) | 11 (11.0) | 5 (7.5) | 5 (8.5) | 7 (18.4) | 2 (8.0) | 1 (4.4) | 6 (27.3) |  | |
| Diabetes | 16183 (20.8) | 300 (21.3) | 134 (18.2) | 71 (27.8) | 49 (27.7) | 19 (19.0) | 15 (22.4) | 14 (23.7) | 9 (23.7) | 5 (20.0) | 3 (13.0) | 3 (13.6) |  | |
| Renal Disease | 9,649 (12.4) | 284 (20.2) | 116 (15.8) | 58 (22.8) | 68 (38.4) | 15 (15.0) | 12 (17.9) | 17 (28.8) | 6 (15.8) | 4 (16.0) | 8 (34.8) | 4 (18.2) |  | |
| Pulmonary disease | 5,927 (7.6) | 161 (11.4) | 89 (12.1) | 34 (13.3) | 25 (14.1) | 7 (7.0) | 2 (3.0) | 8 (13.6) | 3 (7.9) | 1 (4.0) | 3 (13.0) | 2 (9.1) |  | |
| CVD | 4,227 (5.4) | 103 (7.3) | 53 (7.2) | 19 (7.5) | 15 (8.5) | 8 (8.0) | 2 (3.0) | 5 (8.5) | 1 (2.6) | 0 | 2 (8.7) | 3 (13.6) |  | |
| Cancer | 4,781 (6.1) | 67 (4.7) | 29 (3.9) | 13 (5.1) | 8 (4.5) | 4 (4.0) | 6 (9.0) | 1 (1.7) | 0 | 3 (12.0) | 4 (17.4) | 0 |  | |
| Obesity | 3119 (4.0) | 47 (3.3) | 23 (3.1) | 5 (2.0) | 7 (3.95) | 6 (6.0) | 0 (0.0) | 5 (8.5) | 2 (5.3) | 0 | 0 | 0 |  | |
| PVD | 1,754 (2.3) | 40 (2.8) | 22 (3.0) | 7 (2.8) | 3 (1.7) | 5 (5.0) | 4 (6.0) | 1 (1.7) | 0 | 0 | 0 | 0 |  | |
| Dementia | 1,326 (1.7) | 28 (2.0) | 13 (1.8) | 9 (3.5) | 3 (1.7) | 0 | 1 (1.5) | 2 (3.4) | 0 | 0 | 0 | 1 (4.6) |  | |
| Peptic ulcer disease | 871 (1.1) | 34 (2.4) | 24 (3.3) | 4 (1.6) | 2 (1.1) | 3 (3.0) | 1 (1.5) | 1 (1.7) | 2 (5.3) | 0 | 0 | 0 |  | |
| Liver disease | 443 (0.6) | 16 (1.2) | 5 (0.7) | 2 (0.8) | 3 (1.7) | 2 (2.0) | 0 | 0 | 3 (7.9) | 0 | 1 (4.4) | 0 |  | |
| AIRD auto-immune rheumatic disease; RA rheumatoid arthritis; PMR polymyalgia rheumatic; SNV systemic necrotising vasculitis; SLE systemic lupus erythematosus; SSc systemic sclerosis; AS ankylosing spondylitis  PsA psoriatic arthritis; DMPM dermatomyositis/polymyositis; MCTD mixed connective tissue disease; SjS Sjogren’s syndrome | | | | | | | | | | | | | |  |
