# Supplementary material for: Increased 30-day and 1-year mortality rates and lower coronary revascularisation rates following acute myocardial infarction in patients with autoimmune rheumatic disease
Source: Arthritis Res Ther. 2015 Feb 27;17(1):38. doi: 10.1186/s13075-015-0552-2 (PMC4372281; doi:10.1186/s13075-015-0552-2)
Supplement: Additional file 3: — Results of matched case–control (1:5 ratio) analysis. Table S1 Patient demographic and clinical factors, based on case–control analysis. Table S2 Outcomes in patients with versus without autoimmune rheumatic disease (AIRD) who experienced a first MI between 1 July 2001 and 30 June 2007, based on case–control analysis. [file 13075_2015_552_MOESM3_ESM.docx]

**Appendix 3 – Results of matched Case Control (ratio 1:5) analysis (web only)**

**Table S1 – Patient Demographic and Clinical Factors, based on Case-Control analysis (web only)^**

|  | **AIRD** | **Non-AIRD** | | **p-value** |
| --- | --- | --- | --- | --- |
| Patients, n | 1,409 | 7,044 | |  |
| Age, med (IQR) | 77 (68 - 83) | 77 (68 - 83) | | - |
| Female, n (%) | 894 (63.5) | 4,469 (63.4) | | - |
| Indigenous^$^, n (%) | 6 (2.0) | 30 (2.3) | | 0.785 |
| Married / de-facto^$^, n (%) | 137 (46.3) | 713 (55.4) | | 0.009 |
| High accessibility to services^#&^, n (%) | 1,232 (87.8) | 6,144 (87.7) | | 0.925 |
| Lowest Quartile of SEIFA-IRSD, n (%) | 190 (13.5) | 993 (14.1) | | 0.547 |
| Elective admission*, n (%) | 213 (19.1) | 1,059 (18.5) | | 0.603 |
| Public Hospital Admission*, n (%) | 709 (63.7) | 3,817 (66.6) | | 0.062 |
| Comorbidities at Index Admission, n (%) | | | |  |
| Hypertension | 579 (41.1) | 3,354 (47.6) | | <0.001 |
| Arrhythmia | 437 (31.0) | 2,210 (31.4) | | 0.791 |
| Congestive Heart Failure | 511 (36.3) | 2,149 (30.5) | | <0.001 |
| Diabetes | 300 (21.3) | 1,611 (22.9) | | 0.196 |
| Smoker | 168 (11.9) | 1,079 (15.3) | | 0.001 |
| Hypercholesterolaemia | 143 (10.2) | 1,050 (14.9) | | <0.001 |
| Renal Disease | 284 (20.2) | 920 (13.1) | | <0.001 |
| Pulmonary disease | 161 (11.4) | 615 (8.7) | | 0.001 |
| Cancer | 67 (4.7) | 486 (6.9) | | 0.003 |
| Cerebral Vascular Accident | 103 (7.3) | 442 (6.3) | | 0.149 |
| Obesity | 47 (3.3) | 299 (4.2) | | 0.116 |
| Paraplegia | 51 (3.6) | 242 (3.4) | | 0.730 |
| Peripheral Vascular Disease | 40 (2.8) | 161 (2.3) | | 0.213 |
| Dementia | 28 (2.0) | 129 (1.8) | | 0.692 |
| Peptic ulcer disease | 34 (2.4) | 83 (1.2) | | <0.001 |
| Liver disease | 16 (1.2) | 38 (0.5) | | 0.010 |
| HIV | 1 (<0.1) | 0 (-) | | - |
| *^$^ Available for WA data only, n=1,580* | | | |  |
| *^#^ Based on ARIA Code = 1, Major Cities, Highly Accessible* | | |  |  |
| *& Missing data for ARIA in VIC data (n=444)* | | | |  |
| ** Available for VIC data only, n=6,845*  *^ Cases matched to controls based on age and gender* | | | |  |

**Table S2 – Outcomes in patients with and without auto-immune rheumatic disease (AIRD) who experienced a first MI between 1 July 2001 and 30 June 2007, based on Case-Control analysis (web only)^**

| **Variable** | **Non-AIRD (n=7,044)** | **AIRD (n=1,409)** |  |  |
| --- | --- | --- | --- | --- |
|  |  |  |  |  |
|  |  |  | **Crude OR (95% CI)** | **Adjusted* OR (95% CI)** |
|  |  |  |  |  |
| 30-day mortality, all cause | 1,076 (15.3) | 301 (21.4) | 1.51 (1.31 – 1.74) | 1.42 (1.22 – 1.65) |
| 30-day mortality, cardiovascular | 942 (13.4) | 266 (18.9) | 1.51 (1.30 – 1.75) | 1.42 (1.21 – 1.66) |
| 12-month mortality, all cause | 1,852 (26.3) | 544 (38.6) | 1.76 (1.56 – 1.99) | 1.76 (1.54 – 2.01) |
| 12-month mortality, cardiovascular | 1,580 (22.4) | 464 (32.9) | 1.70 (1.50 – 1.92) | 1.66 (1.45 – 1.90) |
|  |  |  |  |  |

^ Cases matched to controls based on age and gender

* Adjusted for ARIA, SEIFA and Charlson comorbidities OR=odds ratio
